# Cincho-Quinine

**Published:** 1875-10

**Authors:** A. G. Smythe

**Affiliations:** Baldwyn, Miss.


*Cincho-Quinine.*

There is a card in the April number of this journal, calling the attention of the faculty to two analyses of cincho-quinine.

Now, in order that all the facts in the case may be fairly presented to the readers of the RECORD, I wish to call the attention of the editors, as well as that of the medical public, to an article in a late (April) number of the *American Journal of Pharmacy*, upon the chemical examination of the article in question, by Emil Scheffer and C. Lewis Diehl, of Louisville, Kentucky. The length of the article referred to precludes its reproduction in this journal; but what I wish to do is to call attention to the conclusion of that article, which is as follows:

"The results of three series of experiments—qualitative and quantitative—may be summed up in the following:

"1. Cincho-quinine is composed mainly of cinchonia, a considerable portion of which is in combination with sulphuric acid, and is, therefore, sulphate of cinchonia.

"2. It contains less than 1 (one) per cent. of the alkaloid quinia, which exists either in alkaloid or as sulphate.

"3. It contains less than 5 (five) per cent. of the alkaloid quinidia, which exists either as alkaloid or as sulphate.

"4. If it contains any cinchonidia at all, this can be present only in very small quantities, since the residue, remaining after exhausting precipitated cincho-quinine with ether, did not contain it, and it could, therefore, have been contained only in the ethereal extraction, in which we did not search for it.

"5. It contains traces of sulphate of ammonium, and is, therefore, precipitated from combination with sulphuric acid by ammonia.

"6. It is not an alkaloid representative of cinchona bark."

There are several other points in the latter part of the article quoted, which it might be well to direct attention to, but the sixth and last assertion is of sufficient importance to demand a searching investigation of the subject.

There are grave doubts in the minds of a large and respectable number of practitioners as to the therapeutic value of cincho-quinine, and it is doubtless the sincere desire of every member of the profession, whose attention has been called to this subject, that the true value of the article in question be definitely settled.

In conclusion, I would suggest that if cincho-quinine is a true representative of cinchona bark, is a cheaper and more pleasant remedy than the preparations heretofore in use, and can establish the character claimed by its friends, why, let us have it; but if, as is asserted in the article quoted, it is not what it is represented to be, and is a worthless nostrum, let it be consigned to oblivion; the sooner the better. A. G. SMYTHE, M. D.

*Baldwyn, Miss., April* 29, 1875.

